# Clinical Characteristics, Care Trajectories and Mortality Rate of SARS-CoV-2 Infected Cancer Patients: A Multicenter Cohort Study

**DOI:** 10.3390/cancers13194749

**Published:** 2021-09-23

**Authors:** Marc-Antoine Benderra, Ainhoa Aparicio, Judith Leblanc, Demian Wassermann, Emmanuelle Kempf, Gilles Galula, Mélodie Bernaux, Anthony Canellas, Thomas Moreau, Ali Bellamine, Jean-Philippe Spano, Christel Daniel, Julien Champ, Florence Canouï-Poitrine, Joseph Gligorov, on Behalf of the AP-HP/Universities/INSERM COVID-19 Research Collaboration, Cancer AP-HP Group COVID-19 Task Force, AP-HP Covid CDW Initiative (ACCI)

**Affiliations:** 1 Medical Oncology Department, AP-HP Greater Paris University Hospital, Tenon Hospital, 75020 Paris, France; gilles.galula@aphp.fr (G.G.); joseph.gligorov@aphp.fr (J.G.); 2 Institut Universitaire de Cancérologie AP-HP Sorbonne Université, Sorbonne Université, 75006 Paris, France; Anthony.canellas@aphp.fr (A.C.); jean-philippe.spano@aphp.fr (J.-P.S.); 3 Alliance Pour la Recherche en Cancérologie, 75020 Paris, France; 4 Clinical Research Platform of East of Paris, AP-HP greater Paris University Hospital, Sorbonne Université Hospitals, 75020 Paris, France; ainhoa.aparicio-ext@aphp.fr (A.A.); judith.leblanc@aphp.fr (J.L.); 5 Sorbonne Université, INSERM, Institut Pierre Louis d’Épidémiologie et de Santé Publique (IPLESP), 75011 Paris, France; 6 Institut National de Recherche en Informatique et en Automatique, INRIA Saclay Île-de-France, 78150 Palaiseau, France; demian.wassermann@inria.fr; 7 Medical Oncology Department, AP-HP Greater Paris University Hospital, Henri-Mondor Hospital, 94000 Creteil, France; Emmanuelle.kempf@aphp.fr; 8 Cancer Group, AP-HP Greater Paris University Hospital, 75004 Paris, France; 9 AP-HP Greater Paris University Hospital, DST, 75012 Paris, France; melodie.bernaux@aphp.fr; 10 Pneumology Department, AP-HP Greater Paris University Hospital, Tenon Hospital, 75020 Paris, France; 11 Université Paris-Saclay, Inria, CEA, 91120 Palaiseau, France; thomas.moreau@inria.fr; 12 AP-HP Greater Paris University Hospital, DSI-WIND, 75006 Paris, France; ali.bellamine@aphp.fr (A.B.); christel.daniel@aphp.fr (C.D.); 13 Medical Oncology Department, AP-HP Greater Paris University Hospital, Pitie-Salpetriere Hospital, Paris 75013, France; 14 Institut Pierre Louis d'Épidémiologie et de Santé Publique (iPLESP), Institut National de la Santé et de la Recherche Médicale (Inserm) 1136, Sorbonne Université, Paris 75013, France; 15 INSERM UMR_S 1142, LIMICS, Sorbonne Université, 75006 Paris, France; 16 Institut National de Recherche en Informatique et en Automatique, INRIA Sophia-Antipolis—ZENITH Team, LIRMM, Montpellier 75646, France; julien.champ-ext@aphp.fr; 17 Public Health Department, APHP Greater Paris University Hospital, Henri-Mondor Hospital, 94000 Creteil, France; Florence.canoui-poitrine@aphp.fr; 18 IMRB-INSERM U955, CEpiA Team, Université Paris Est Créteil, 94000 Créteil, France; 19 CRSA-INSERM U938, Cancers Biology and Treatment Team, Sorbonne Université, 75012 Paris, France

**Keywords:** SARS-CoV-2 infection, hospitals, cancer, mortality rate, multicenter study

## Abstract

**Simple Summary:**

COVID-19 may be more frequent and more severe in cancer patients than in other individuals. Our aims were to assess the rate of COVID-19 in hospitalized cancer patients, to describe their demographic characteristics, clinical features and care trajectories, and to assess the mortality rate. A total of 1148 hospitalized patients were included. The mortality rate was 33%. In multivariate analysis, mortality-related factors were male sex, advanced age, more than two comorbidities, C-reactive protein >20 ng/mL, primary brain tumors and lung cancer. Risk of dying was lower among patients with metabolic comorbidities.

**Abstract:**

Background: COVID-19 may be more frequent and more severe in cancer patients than in other individuals. Our aims were to assess the rate of COVID-19 in hospitalized cancer patients, to describe their demographic characteristics, clinical features and care trajectories, and to assess the mortality rate. Methods: This multicenter cohort study was based on the Electronic Health Records of the Assistance Publique-Hôpitaux de Paris (AP-HP). Cancer patients with a diagnosis of COVID-19 between 3 March and 19 May 2020 were included. Main outcome was all-cause mortality within 30 days of COVID-19 diagnosis. Results: A total of 29,141 cancer patients were identified and 7791 (27%) were tested for SARS-CoV-2. Of these, 1359 (17%) were COVID-19-positive and 1148 (84%) were hospitalized; 217 (19%) were admitted to an intensive care unit. The mortality rate was 33% (383 deaths). In multivariate analysis, mortality-related factors were male sex (aHR = 1.39 [95% CI: 1.07–1.81]), advanced age (78–86 y: aHR = 2.83 [95% CI: 1.78–4.51] vs. <66 y; 86–103 y: aHR = 2.61 [95% CI: 1.56–4.35] vs. <66 y), more than two comorbidities (aHR = 2.32 [95% CI: 1.41–3.83]) and C-reactive protein >20 ng/mL (aHR = 2.20 [95% CI: 1.70–2.86]). Primary brains tumors (aHR = 2.19 [95% CI: 1.08–4.44]) and lung cancer (aHR = 1.66 [95% CI: 1.02–2.70]) were associated with higher mortality. Risk of dying was lower among patients with metabolic comorbidities (aHR = 0.65 [95% CI: 0.50–0.84]). Conclusions: In a hospital-based setting, cancer patients with COVID-19 had a high mortality rate. This mortality was mainly driven by age, sex, number of comorbidities and presence of inflammation. This is the first cohort of cancer patients in which metabolic comorbidities were associated with a better outcome.

## 1. Introduction

At the onset of the COVID-19 pandemic, early reports identified multiple factors associated with worse outcomes of COVID-19 patients, including advanced age, male sex and comorbidities such as cardio-metabolic disorders, respiratory diseases and cancer [1,2]. Data from China suggested that patients with active or previous cancer were at higher risk of SARS-CoV-2 infection [3] and death [4]. On this basis, national and international guidelines recommended caution regarding the continuation of cancer screening, cancer treatments and physical follow-up of cancer patients [5].

Previous data suggest that the type of primary malignancy might be associated with COVID-19 prognosis. The mortality rates for COVID-19 vary widely among cancer patients, ranging from 9% to 33%, and no clear association has been found between anticancer therapies, tumor characteristics and COVID-19 mortality. The spread of the pandemic and its impact is very heterogeneous across and within different countries. These disparities have motivated epidemiological reports from several countries and institutions, to better explain the differences in mortality rates observed across studies. Better knowledge of the risk factors for COVID-19 mortality in cancer patients is crucial and might help to optimize the management of patients in the future.

In France, the Paris metropolitan area was one of the most affected regions during the first wave of the COVID-19 pandemic. 

The aims of the current study were to describe the demographic and clinical characteristics of hospitalized COVID-19-positive cancer patients during the first wave of the pandemic in this region, to determine the in-hospital mortality rate, to assess the care trajectories and to identify prognostic factors.

## 2. Materials and Methods

### 2.1. Study Design

This was a retrospective, multicenter cohort study based on Electronic Health Record (EHR) data collected prospectively in the Assistance Publique-Hôpitaux de Paris (AP-HP) Information System and collated in the AP-HP Health Data Warehouse (EDS AP-HP). The AP-HP is the main public health establishment and university hospital center in the Paris metropolitan area, providing hospital care for a population of 6.7 million individuals. The study was approved by the Institutional Review Board (IRB: 00011591) on 16 April 2020. The database was authorized by the National Freedom and Informatics Commission (CNIL Number: 1980120). 

### 2.2. Setting

AP-HP is the largest hospital system in Europe and one of the largest worldwide. The 39 hospitals of AP-HP (List S2) provide nearly 6 million hospital stays and outpatient visits, including 1.5 million emergency visits each year. More than 60,000 patients are treated annually or followed for their cancer in AP-HP centers, including about 40,000 new cases. A specific AP-HP COVID clinical data repository (COVID CDR) database was derived from the institutional clinical data repository, which has integrated administrative and clinical data from AP-HP centers since 2013 [6]. The COVID CDR includes clinical data for all patients tested or diagnosed with COVID-19 since 1 January 2020, and collected during inpatient or outpatient visits. The clinical data is interoperable and standardized using the Observational Medical Outcomes Partnership Common Data Model of the Observational Health Data Science and Informatics distributed data network and international terminologies.

### 2.3. Study Population

The study population consisted of all patients, including children, tested for SARS-CoV-2 and hospitalized during the first wave of the pandemic in one of the AP-HP centers. The diagnosis of cancer was determined using the codes listed in the International Statistical Classification of Diseases and Related Health Problems 10th Revision (ICD-10) (main diagnosis, related diagnosis or associated diagnosis during hospital stay), corresponding either to cancer or to cancer-related procedures including tumor surgery, radiotherapy and chemotherapy (Table S1). For each patient, history of cancer during the last 8 years was taken into account in the CDR. 

### 2.4. Procedures

Demographic characteristics and in-hospital mortality were collected. COVID-19 infection was confirmed by a positive RT-PCR test for SARS-CoV-2. Cancer diagnosis, cancer location and metastatic status were identified using ICD-10 codes. Coexisting conditions (hypertension, coronary heart disease, congestive heart failure, cardiac arrhythmia, diabetes, hyperlipidemia, obesity, chronic obstructive pulmonary disease (COPD), chronic kidney disease), and smoking were identified using ICD-10 codes (main diagnosis, related diagnosis, associated diagnosis) during the study period or during previous hospitalizations (Table S1), except for obesity retrieved in the previous 12 months. Laboratory parameters were collected at the closest date to the SARS-CoV-2 test, within a 5-day window. Cancer treatment within the 3 months preceding COVID-19 diagnosis and treatment including admission to an intensive care unit (ICU), mechanical ventilation and drug therapy were retrieved from the database. Manual reading of the in-hospital reports for each included patient was performed to extract the relevant information. 

### 2.5. Outcomes

The primary outcome was all-cause mortality within 30 days of the diagnosis of COVID-19. Secondary outcomes were clinical and tumor characteristics, care trajectories including hospitalization in an ICU, and discharge.

### 2.6. Statistical Analysis

Baseline demographic, cancer-related and non-cancer-related characteristics are described. Categorical variables are described as numbers and percentages, and continuous variables as medians and interquartile range. The mortality rate was calculated up until day 30. 

Univariate Cox proportional hazards analyses were conducted. The proportional hazards assumption was tested using Schoenfeld residuals and the Grambsch-Therneau test. Variables associated with the main endpoint at *p* < 0.20 were retrieved for multivariate analysis. Interactions were tested using the Wald test. Forward and backward stepwise regression was performed to reduce the null model. 

The care trajectories of the patients are described according to the initial care (hospitalization or ICU hospitalization). 

The threshold for statistical significance was set at *p* < 0.05. No imputation was performed. All tests were two-tailed and all statistical analyses were performed using R^®^ 2.4.3 and Python^®^ 2.4.3. 

Reporting was conducted in accordance with observational, routinely-collected data (RECORD) guidelines [7].

## 3. Results

### 3.1. Characteristics of the Study Population

**Figure 1:**
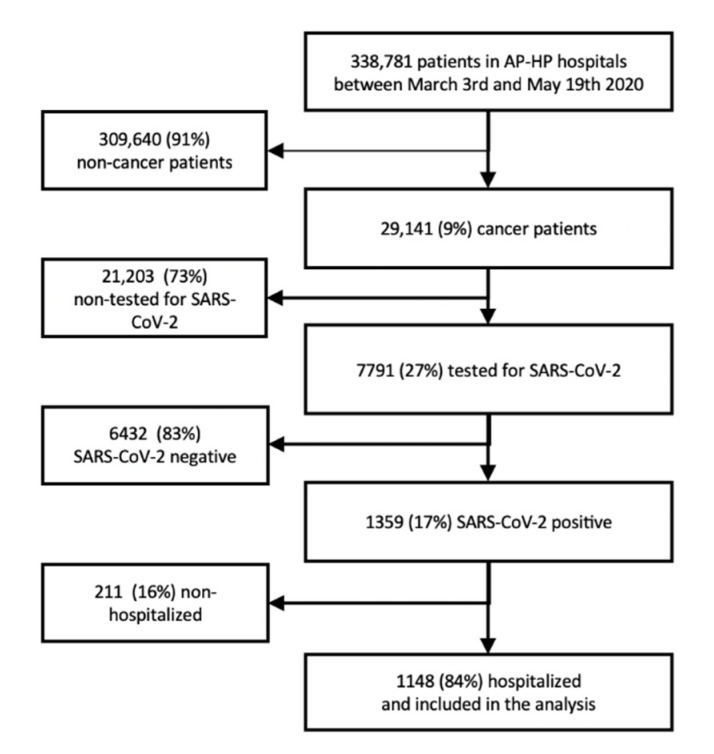
Flow-chart of the study.

Between 3 March 2020 and 19 May 2020, 338,781 patients were referred to an AP-HP hospital (outpatient, or admission), 29,141 of whom either had an active tumor or a history of cancer (Figure 1). Among them, 7791 (27%) underwent a SARS-CoV-2 RT-PCR test. A total of 1359 patients (17%) had a confirmed COVID-19 diagnosis. Among these, 1148 (84%) were hospitalized and included in the analysis. The main baseline features of the hospitalized patients are summarized in Table 1. Median age was 76-years and 41% of the patients were women. Overall, 50% had hypertension, 34% a history or current coronary heart disease or congestive heart failure, 26% had a history or current cardiac arrhythmia, 31% diabetes mellitus, 27% chronic kidney disease and 22% had COPD. Approximately 11% of the patients were former or current smokers, 8% were obese and 15% had hyperlipidemia. In total, 464 patients (40%) had three or more comorbidities.

The cancer-related characteristics of the study population are shown in Table 2. Hematologic malignancies (*n* = 264) represented 23% of all cancers. For solid tumors, the most frequent types of tumors were urinary tract cancers (*n* = 191, 17%), digestive cancers (*n* = 129, 11%) and breast cancers (*n* = 99, 9%). Overall, 318 patients (28%) had metastatic disease. Overall, 217 (19%) patients were admitted to an ICU. The treatments administered for COVID-19 are shown in Table S2.

### 3.2. Factors Associated with All-Cause Mortality 

**Figure 2:**
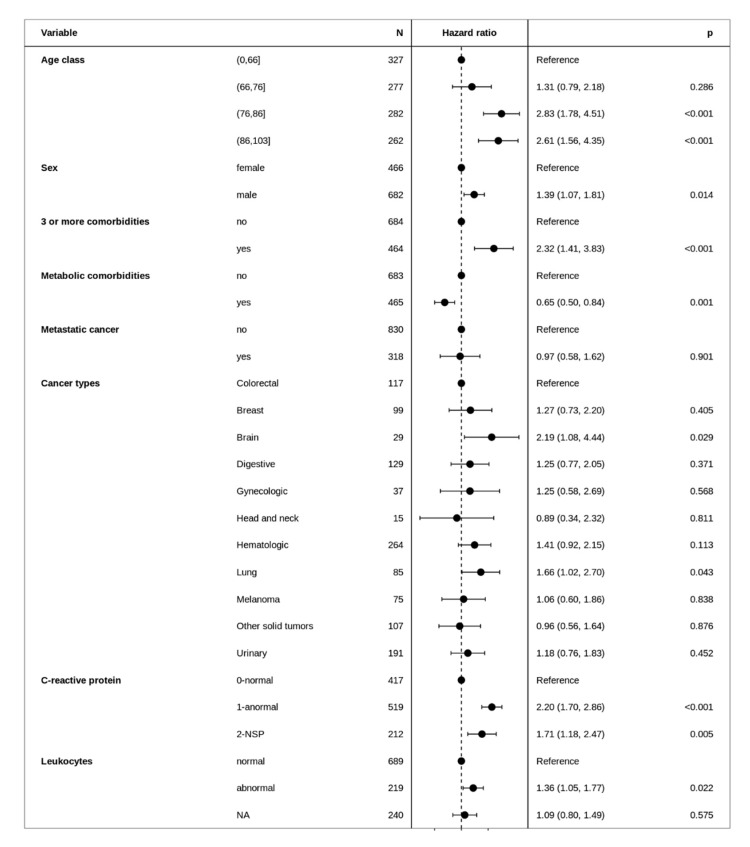
Forest plot of the multivariate analysis of factors associated with all-cause mortality within 30 days of COVID-10 diagnosis. NA/NSP: Not Available.

The all-cause mortality rate within 30 days of a COVID-19 diagnosis was 33%, accounting for 383 deaths. In univariate analysis (Table 1 and Table 2), patients who died were older, more likely to be male, more frequently had coronary heart disease or congestive heart failure, cardiac arrhythmia, COPD, chronic kidney disease, elevated leukocyte counts and C-reactive protein. In multivariate analysis, older patients (aHR = 2.83 [95% CI: 1.78–4.51] for 76–86-years-old; aHR = 2.61 [95% CI: 1.56–4.35] for 86–103-years-old) were at higher risk of dying than patients <66-years-old. Male sex (aHR = 1.39 [95% CI: 1.07–1.81]), more than two comorbidities (aHR = 2.32 [95% CI: 1.41–3.83]), leukocytes ≥10 × 10^9^/L (aHR = 1.36 [95% CI: 1.05–1.77]) and C-reactive protein >20 ng/mL (aHR = 2.20 [95% CI: 1.70–2.86]) were also independently associated with mortality (Figure 2). Patients with brain tumors (aHR = 2.19 [95% CI: 1.08–4.44]) and lung tumors (aHR = 1.66 [95% CI: 1.02–2.70]) had a higher risk of mortality. The risk of mortality was lower among patients with metabolic comorbidities (obesity, hyperlipidemia or diabetes mellitus) (aHR = 0.65 [95% CI: 0.50–0.84]). 

### 3.3. Care Trajectories

**Figure 3:**
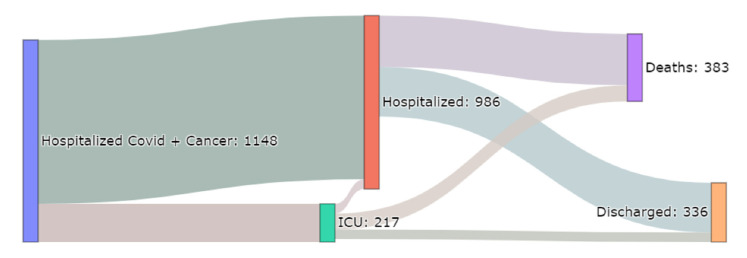
Flow of trajectories of hospitalized cancer patients diagnosed for COVID-19 at AP-HP up to 20 May 2020.

The care trajectories are shown in Figure 3. Among the 217 patients hospitalized in an ICU, 91 (42%) died in the ICU, 17 (8%) were in an ICU at 30-day follow-up, and 109 (50%) were discharged. Among the 383 deaths, 292 (76%) patients were hospitalized outside an ICU. 

## 4. Discussion

This French multicenter, hospital-based study gathered medical data for more 300,000 patients during the study period, using an EHR Data warehouse. Amongst the cancer patients who were hospitalized, the mortality rate from COVID-19 was 33%. The main risk factors for death were advanced age, male sex, number of comorbidities, tumor type (brain and lung tumors) and presence of biological inflammation. 

The mortality rate observed in our study was similar to that observed in a UK study [8] and another French study [9], which reported a mortality rate of 28% and 29%, respectively. Nevertheless, it is important to stress that the rate of ICU admission differs from one country to another: 19% in our study population, which is the highest rate. Despite the constraints encountered during the first COVID-19 wave, this shows that the healthcare settings participating in this study were able to implement the necessary means and provide additional intensive care beds.

Compared to other multicenter cohort studies [8,10], our study population was markedly older with more comorbidities, including cardio-metabolic and pulmonary-associated diseases (Table 3). Tumor site distribution in our cohort differed from that in Chinese and US cohorts [10,11], which reported more breast cancers [8], but was similar to that in a UK study [12]. Uncertainty regarding metastatic status definition exists in other studies as hematological malignancies may be included, preventing any comparison. 

The advanced age of our study population, including inpatients, might account for the high case-fatality rate observed. It is now well known that age >65-years is a strong prognostic factor for COVID-19 [11]. It is also known that older cancer patients have an increased risk of comorbidities and frailty, which are independently associated with higher mortality rates [13,14]. Since these comorbidities are competing risks of death in older patients with cancer, it is challenging to demonstrate whether COVID-19-related death is linked to the underlying cardio-metabolic or pulmonary comorbidity, or to the cancer and/or its treatments. In some COVID-19 cancer patients, age may also be a criterion for treatment limitations, particularly in the case of severe comorbidities [15,16]. In our study, there was an association between number of comorbidities and mortality. Nevertheless, all the comorbidities did not have the same prognostic value. Metabolic comorbidities (including obesity, diabetes mellitus and hyperlipidemia) were associated with decreased mortality. A large number of retrospective studies have suggested that obesity increases the risk of death associated with COVID-19 [15,16,17]. Nevertheless, in the French CORONADO trial [18] evaluating the risk of mechanical ventilation or death by day 7 of hospital admission, no relationship between obesity and mortality was observed in patients >75-years-old. The existence of an obesity paradox phenomenon might be explored in older patients with COVID-19 [19]. Primary data support a greater risk of more severe COVID-19 outcomes in patients with type 2 diabetes [20]. However, in some large studies, type 2 diabetes was not associated with COVID-19 mortality in hospitalized patients [21]. Cholesterol metabolism has also been the subject of numerous studies and has been identified as a potential target for modification of viral infectivity. In preclinical studies, higher levels of low-density lipoproteins may be associated with protection against ICU admission and mortality from sepsis [22]. Moreover, a large retrospective study demonstrated that the in-hospital use of statins was associated with a lower risk of mortality [23]. In our study, the use of statins could be a cofounding factor. Finally, the association between metabolic comorbidities and a lower risk of mortality could reflect the absence of weight loss induced by COVID-19 infection, which is a major prognostic factor in cancer patients [24], especially in those with obesity. In a recent review, Drucker et al. highlighted the controversies and unresolved questions that exist in metabolic aspects of SARS-Cov-2 induced pathophysiology [25]. 

In our population, patients with brain or lung tumors had a poor outcome, with up to 43% mortality in patients with lung cancer. An high case-fatality rate of around 33% was also described in recent studies [26,27]. In France, it will be interesting to evaluate the mortality rates of cancer patients during subsequent waves of COVID-19.

In our study, we found no association between the administration of anticancer treatments in the previous 3 months and mortality. These data, although incomplete (20% missing data for anticancer treatments), support current recommendations encouraging clinicians not to undertreat cancer patients [28].

The main strengths of our study are the very large study population and the inclusion of exclusively hospitalized patients, precluding the risk of selection bias and the risk of a biased case-fatality rate. Secondly, the size of the study population led to accurate estimations. However, the study has several limitations. First, ICD coding habits may differ among centers leading to a possible classification bias. Secondly, ICD coding may underestimate some comorbidities or behaviors, particularly obesity or smoking. Neuro-language processing in textual EHR may improve the reliability of these measures. Finally, this study, similar to other previous studies, was hospital-based, therefore, extrapolation of our results to all cancer patients in an ambulatory setting needs to be confirmed. 

## 5. Conclusions

The high COVID-19 mortality rate observed amongst cancer patients was mainly driven by advanced age, male sex and number of cardiovascular and respiratory comorbidities. Metabolic comorbidities may be associated with a lower risk of mortality. The high case-fatality rate strengthens the need to prevent the risk of SARS-CoV-2 infection in patients with active or previous cancer, and to carefully balance the risk-benefit ratio of cancer treatments regarding short-term cancer evolution versus risk of COVID-19. 

## Figures and Tables

**Table 1:** Patients’ characteristics and coexisting conditions among cancer patients with confirmed COVID-19. * Groups were compared using Pearson chi-square or Student T test as appropriate. ** metabolic comorbidities include diabetes mellitus, hyperlipidemia or obesity.

Patients’ Characteristics	All Patients (*N* = 1148)	Patients Who Survived (*N* = 765)	Patients Who Died (*N* = 383)	*p* Value *
Sex (%)				
Female	466 (40.6)	337 (44.1)	129 (33.7)	<0.001
Age, years				
Median (range)	75.5 (4–103)	74.0 (8–103)	79.0 (4–100)	<0.001
<66	327 (28.5)	246 (32.2)	81 (21.1)	
66–75	277 (24.1)	192 (25.1)	85 (22.2)	
76–85	282 (24.6)	162 (21.2)	120 (31.3)	
≥86	262 (22.8)	165 (21.6)	97 (25.3)	
Comorbidities (%)				
Hypertension	575 (50.1)	371 (48.5)	204 (53.3)	0.144
Coronary heart disease or congestive heart failure	385 (33.5)	224 (29.3)	161 (42)	<0.0001
Cardiac arrythmia	294 (25.6)	170 (22.2)	124 (32.4)	<0.0001
Diabetes mellitus	351 (30.6)	231 (30.2)	120 (31.3)	0.745
Hyperlipidemia	172 (15.0)	125 (16.3)	47 (12.3)	0.083
Obesity	96 (8.4)	72 (9.4)	24 (6.3)	0.089
Metabolic comorbidities **	465 (40.5)	318 (41.6)	147 (38.4)	0.33
Chronic obstructive pulmonary disease	251 (21.9)	148 (19.3)	103 (26.9)	0.004
Smoker or history of smoking	122 (10.7)	76 (10.1)	46 (12.1)	0.352
Chronic kidney disease	314 (27.4)	190 (24.8)	124 (32.4)	0.008
Thrombosis	195 (17.0)	127 (16.6)	68 (17.8)	0.684
Number of comorbidities (%)				
0	256 (22.3)	181 (23.7)	75 (19.6)	0.003
1	216 (18.8)	163 (21.3)	53 (13.8)	
2	212 (18.5)	137 (17.9)	75 (19.6)	
≥3	464 (40.4)	284 (37.1)	180 (47)	0.002
Laboratory findings (%)				
Leukocytes				
<10 × 10^9^/L	689 (60)	481(62.9)	208 (54.3)	<0.001
≥10 × 10^9^/L	219 (19.1)	123 (16.1)	96 (25.1)	
Missing data	240 (20.9)	161 (21)	79 (20.6)	
Lymphocytes				
<1.5 × 10^9^/L	705 (61.4)	468 (61.2)	237 (61.9)	0.965
≥1.5 × 10^9^/L	184 (16)	124 (16.2)	60 (15.6)	
Missing data	259 (22.6)	173 (22.6)	86 (22.5)	
Platelets				
<150 × 10^9^/L	250 (21.8)	165 (21.6)	85 (22.2)	0.966
≥150 × 10^9^/L	658 (57.3)	439 (57.4)	219 (57.2)	
Missing data	240 (20.9)	161 (21)	79 (20.6)	
C-reactive protein				
<20 mg/L	417 (36.3)	321 (41.9)	96 (25)	<0.001
≥20 mg/L	519 (45.2)	302 (39.5)	217 (56.7)	
Missing data	212 (18.5)	142 (18.6)	70 (18.3)	

**Table 2:** Tumor characteristics among patients with cancer and confirmed COVID-19. * Groups were compared excluding missing data and using Pearson chi-square or Student T test as appropriate.

Cancers’ Characteristics	All Patients (*N* = 1148)	Patients Who Survived (*N* = 765)	Patients Who Died (*N* = 383)	*p* Value *
Tumor stage				
Metastatic	318 (27.7)	204 (26.7)	114 (29.8)	0.3
Tumor type (%)				
Hematologic	264 (23)	171 (22.4)	93 (24.3)	0.094
Digestive	129 (11.2)	89 (11.6)	40 (10.4)	
Urologic	191 (16.6)	117 (15.3)	74 (19.3)	
Gynecologic	37 (3.2)	24 (3.1)	13 (3.4)	
Pulmonary	85 (7.4)	48 (6.3)	37 (9.7)	
Breast	99 (8.6)	75 (9.8)	24 (6.3)	
Primary Central Nervous System	29 (2.5)	18 (2.4)	11 (2.9)	
Head and neck	15 (1.3)	9 (1.2)	6 (1.6)	
Melanoma of the skin	75 (6.5)	52 (6.8)	23 (6)	
Other solid tumors	107 (9.3)	81 (10.6)	26 (6.8)	
Cancer treatment in the 3 months preceding COVID-19 diagnosis (%)				
Chemotherapy Missing data	216 (18.8) 95 (8.3)	142 (18.6) 60 (7.8)	74 (19.3) 35 (9.1)	0.69
Hormone therapy Missing data	67 (5.8) 97 (8.4)	45 (5.9) 62 (8.1)	22 (5.7) 35 (9.1)	0.837
Immunotherapy Missing data	85 (7.4) 97 (8.4)	50 (6.5) 62 (8.1)	35 (9.1) 35 (9.1)	0.217
Radiotherapy Missing data	26 (2.3) 98 (8.5)	13 (1.7) 62 (8.1)	13 (3.4) 36 (9.4)	0.136
Surgery Missing data	66 (5.7) 144 (12.5)	54 (7.1) 87 (11.4)	12 (3.1) 57 (14.9)	0.009
Targeted treatment Missing data	81 (7.1) 96 (8.4)	53 (6.9) 61 (8)	28 (7.3) 35 (9.1)	0.764

**Table 3:** Main Cohort studies with COVID-19 cancer patients. Abbreviations: NA, Not Available; COPD, chronic obstructive pulmonary disease; ICU, Intensive Care Unit.

Study	Lee et al. [12]	Kuderer et al. [8]	Yang et al. [10]	Lievre et al. [9]	Benderra et al.
Cancer population, n	800	928	205	1289	1148
Region	UK, 55 centers	US, Canada, Spain	China (Hubei), 9 centers	France, 153 centers	France (metropolitan Paris area), 39 centers
COVID-19 definition	RT-PCR	RT-PCR	RT-PCR	RT-PCR or imaging features	RT-PCR
Age, years	69 (59–76)	66 (57–76)	63 (56–70)	67	76 (65–86)
Female gender	349 (44)	459 (49)	109 (53)	113 (49)	466 (41)
Comorbidities—no. (%, on available data)					
Hypertension	247 (31)	NA	67 (33)	529 (46)	575 (50)
Cardiovascular disease	109 (14)	NA	16 (8)	194 (16)	385 (34)
COPD	61 (8)	NA	5 (2)	124 (12)	251 (22)
Diabetes	131 (16)	NA	22 (11)	241 (21)	351 (31)
Cancer type—no. (%, on available data)					
GI	150 (19)	108 (12)	40 (20)	470 (36)	129 (11)
Pulmonary	90 (11)	91 (10)	24 (12)	311 (24)	85 (7)
Breast	102 (13)	191 (21)	40 (20)	173 (13)	99 (9)
Hematological malignancies	169 (22)	204 (22)	22 (11)	0	264 (23)
Metastatic cancer—no. (%)	347 (43)	NA	NA	758 (59)	318 (28)
ICU admission—no. (%, on available data)	53 (7)	132 (14)	30 (15)	110 (10)	217 (19)
Mortality—no. (%)	226 (28)	121 (13)	40 (20)	370 (29)	383 (33)

## Data Availability

The data used in the preparation of this article were obtained from the AP-HP Covid Clinical Data Warehouse (CDW).

## Supplementary Materials

The following are available online at https://www.mdpi.com/article/10.3390/cancers13194749/s1. Table S1: Identification of cancer patients and comorbidities among the AP-HP warehouse, Table S2: Laboratory findings among patients with cancer and confirmed COVID-19, List S1: Supplementary materials of co-authors, List S2: List of the 39 hospitals that make-up Assistance Publique-Hôpitaux de Paris.
