# Supplementary material for: Clinical Characteristics, Care Trajectories and Mortality Rate of SARS-CoV-2 Infected Cancer Patients: A Multicenter Cohort Study
Source: Cancers (Basel). 2021 Sep 23;13(19):4749. doi: 10.3390/cancers13194749 (PMC8507538; doi:10.3390/cancers13194749)
Supplement: Supplementary file 1 [file cancers-13-04749-s001.zip › Supplementary list 1.pdf]

The data used in the preparation of this article were obtained from the AP-HP Covid Clinical Data Warehouse (CDW). As such, the members of the AP-HP Covid CDW initiative (ACCI) contributed to the design and implementation of the database but did not participate in the analysis or writing of this report. A complete listing of ACCI members can be found at: https://eds.aphp.fr/covid-19 and below.
Data used regarding cancer characteristics were obtained from the AP-HP CDW and were previously supplied by AP-HP oncologists. As such, the members of the Groupe Cancer AP-HP contributed to the implementation of the database but did not participate in the analysis or writing of this report. A complete listing of Groupe Cancer AP-HP members can be found in List S2.

List of co-authors

***AP-HP / Universities / Inserm COVID-19 research collaboration, scientific committee**

Philippe-Gabriel Steg, Tabassome Simon, Florence Favrel-Feuillade, Yannick Vacher, Yazdan Yazdanpanah, Helène Espérou, Odile Launay, Dominique Costagliola, Vincent Calvez, Elie Azoulay, Bruno Riou, Claire Hassen Khodja, Sylvie Bastuji-Garin.

[#]Cancer AP-HP Group COVID-19 task force,

Lorea Aguinaga, Jérome Alexandre, Thierry André, Thomas Aparicio, Jalal Assouad, Frederic Batteux, Anne-Sophie Bats, Jean-Baptiste Bachet, Yazid Belkacemi, Chloé Bertolus, Marc-Olivier Bitker, Nicolas Boissel, Jacques Cadranel, Marine Camus Duboc, Stanisals Chaussade, Romain Coriat, Sylvain Choquet, Romain Coriat, Philippe Cornu, Stéphane Culine, Olivier Cussenot, Emile Darai, Jean-Yves Delattre, Hervé Dombret, Bertrand Dousset, Catherine Durdux, Marc Espié, Pierre Fenaux, Gilles Galula, Philippe Giraud, Joseph Gligorov, François Goldwasser, Jérémie Gueye Lefevre, Corinne Haioun, Pascal Hammel, Christophe Hennequin, Olivier Hermine, Florence Huguet, Olivier Lambotte, Pierre Laurent-Puig, Armelle Lavole, Céleste Lebbe, Véronique Leblond, Laurence Leenhardt, Guy Leverger, Jean-Pierre Lotz, Philippe Maingon, Fabrice Menegaux, Mohamad Mohty, Diaretou Ndiaye-Gueye, Stéphane Oudard, Yann Parc, Arnaud Petit, Robert Ratiney, Olivier Rosmorduc, Philippe Ruszniewski, Rémi salomon, Marc Sanson, Olivier Scaton, Gérard Socie, Jean-Philippe Spano, Julien Taieb, Catherine Thieblemont, Isabelle Thomassin, Christophe Tournigand, Catherine Uzan, Jean-Christophe Vaillant, Marie Wislez, Gerard Zalcman, Laurent Zelek

°AP-HP Covid CDW Initiative (ACCI)

Name	Surname	Affiliation
Ancel	Pierre-Yves	APHP Paris University Center
Bauchet	Alain	APHP Saclay University
Beeker	Nathanael	APHP Paris University Center
Benoit	Vincent	WIND Department APHP Greater Paris University Hospital
Bernaux	Mélodie	Strategy and transformation department, APHP Greater Paris University Hospital
Bellamine	Ali	WIND Department APHP Greater Paris University Hospital
Bey	Romain	WIND Department APHP Greater Paris University Hospital
Bourmaud	Aurélie	APHP Paris University North
Bréant	Stéphane	WIND Department APHP Greater Paris University Hospital
Burgun	Anita	Department of Biomedical Informatics, HEGP, APHP Greater Paris University Hospital
Carrat	Fabrice	APHP Sorbonne University
Caucheteux	Charlotte	Université Paris-Saclay, Inria, CEA
Champ	Julien	INRIA Sophia-Antipolis – ZENITH team, LIRMM, Montpellier, France
Cormont	Sylvie	WIND Department APHP Greater Paris University Hospital
Daniel	Christel	WIND Department APHP Greater Paris University Hospital ; UMRS1142 INSERM
Dubiel	Julien	WIND Department APHP Greater Paris University Hospital
Ducloas	Catherine	APHP Paris Seine Saint Denis Universitary Hospital
Esteve	Loic	SED/SIERRA, Inria Centre de Paris
Frank	Marie	APHP Saclay University
Garcelon	Nicolas	Imagine Institute
Gramfort	Alexandre	Université Paris-Saclay, Inria, CEA
Griffon	Nicolas	WIND Department APHP Greater Paris University Hospital UMRS1142 INSERM
Grisel	Olivier	Université Paris-Saclay, Inria, CEA
Guilbaud	Martin	WIND Department APHP Greater Paris University Hospital
Hassen-Khodja	Claire	Direction of the Clinical Research and Innovation, AP-HP
Hemery	François	APHP Henri Mondor University Hospital
Hilka	Martin	WIND Department APHP Greater Paris University Hospital
Jannot	Anne Sophie	Department of Biomedical Informatics, HEGP, APHP Greater Paris University Hospital
Lambert	Jerome	APHP Paris University North
Layese	Richard	APHP Henri Mondor University Hospital
Leblanc	Judith	Clincial Research Unit, Saint Antoine Hospital, APHP Greater Paris University Hospital
Lebouter	Léo	WIND Department APHP Greater Paris University Hospital
Lemaitre	Guillaume	Université Paris-Saclay, Inria, CEA
Leprovost	Damien	Clevy.io
Lerner	Ivan	Department of Biomedical Informatics, HEGP, APHP Greater Paris University Hospital
Levi Sallah	Kankoe	APHP Paris University North
Maire	Aurélien	WIND Department APHP Greater Paris University Hospital
Mamzer	Marie-France	President of the AP-HP IRB
Martel	Patricia	APHP Saclay University
Mensch	Arthur	ENS, PSL University
Moreau	Thomas	Université Paris-Saclay, Inria, CEA

Neuraz	Antoine	Department of Biomedical Informatics, HEGP, APHP Greater Paris University Hospital
Orlova	Nina	WIND Department APHP Greater Paris University Hospital
Paris	Nicolas	WIND Department APHP Greater Paris University Hospital
Rance	Bastien	Department of Biomedical Informatics, HEGP, APHP Greater Paris University Hospital
Ravera	Hélène	WIND Department APHP Greater Paris University Hospital
Rozes	Antoine	APHP Sorbonne University
Salamanca	Elisa	WIND Department APHP Greater Paris University Hospital
Sandrin	Arnaud	WIND Department APHP Greater Paris University Hospital
Serre	Patricia	WIND Department APHP Greater Paris University Hospital
Tannier	Xavier	Sorbonne University
Treluyer	Jean-Marc	APHP Paris University Center
Van Gysel	Damien	APHP Paris University North
Varoquaux	Gael	Université Paris-Saclay, Inria, CEA, Montréal Neurological Institute, McGill University
Vie	Jill-Jênn	SequeL, Inria Lille
Wack	Maxime	Department of Biomedical Informatics, HEGP, APHP Greater Paris University Hospital
Wajsburt	Perceval	Sorbonne University
Wassermann	Demian	Université Paris-Saclay, Inria, CEA
Zapletal	Eric	Department of Biomedical Informatics, HEGP, APHP Greater Paris University Hospital
