# Supplementary material for: Clinical Characteristics, Care Trajectories and Mortality Rate of SARS-CoV-2 Infected Cancer Patients: A Multicenter Cohort Study
Source: Cancers (Basel). 2021 Sep 23;13(19):4749. doi: 10.3390/cancers13194749 (PMC8507538; doi:10.3390/cancers13194749)
Supplement: Supplementary file 1 [file cancers-13-04749-s001.zip › Supplementary list 2.pdf]

Supplementary List 2. List of the 39 hospitals that make-up Assistance Publique-Hôpitaux de Paris.

Hôpital Adélaïde-Hautval, Val-d'Oise, Ile de France
Hôpital Albert-Chenevier, Val-de-Marne, Ile de France
Hôpital Ambroise-Paré, Hauts-de-Seine, Ile de France
Hôpital Antoine-Béclère, Hauts-de-Seine, Ile de France
Hôpital Armand-Trousseau, Paris, Ile de France
Hôpital Avicenne, Seine-Saint-Denis, Ile de France
Hôpital Beaujon, Seine-Saint-Denis, Ile de France
Hôpital Bicêtre, Val-de-Marne, Ile de France
Hôpital Bichat-Claude Bernard, Paris, Ile de France
Hôpital Bretonneau, Paris, Ile de France
Hôpital Broca, Paris, Ile de France
Hôpital Charles-Foix, Val-de-Marne, Ile de France
Hôpital Cochin, Paris, Ile de France
Hôpital Corentin-Celton, Seine-Saint-Denis, Ile de France
Hôpital Emile-Roux, Val-de-Marne, Ile de France
Hôpital Européen Georges-Pompidou, Paris, Ile de France
Hôpital Fernand-Widal, Paris, Ile de France
Hôpital Georges-Clémenceau, Essone, Ile de France
Hôpital Henri-Mondor, Paris, Ile de France
Hôpital Hôtel-Dieu, Paris, Ile de France
Hôpital Jean-Verdier, Seine-Saint-Denis, Ile de France
Hôpital Joffre-Dupuytren, Essone, Ile de France
Hôpital La Roche-Guyon, Val-d'Oise, Ile de France
Hôpital La Rochefoucauld, Paris, Ile de France
Hôpital Lariboisière, Paris, Ile de France
Hôpital Louis-Mourier, Hauts-de-Seine, Ile de France
Hôpital Maritime de Berck, Pas-de-Calais, Hauts-de-France
Hôpital Necker-Enfants Malades, Paris, Ile de France
Hôpital Paul-Brousse, Paris, Ile de France
Hôpital Raymond-Poincaré, Hauts-de-Seine, Ile de France
Hôpital René-Muret, Seine-Saint-Denis, Ile de France
Hôpital Robert-Debré, Paris, Ile de France
Hôpital Rothschild, Paris, Ile de France
Hôpital Saint-Antoine, Paris, Ile de France
Hôpital Saint-Louis, Paris, Ile de France
Hôpital Sainte-Perrine, Paris, Ile de France
Hôpital Tenon, Paris, Ile de France
Hôpital Pitié-Salpétrière, Paris, Ile de France
Hôpital Vaugirard, Paris, Ile de France
