# Supplementary material for: Clinical Characteristics, Care Trajectories and Mortality Rate of SARS-CoV-2 Infected Cancer Patients: A Multicenter Cohort Study
Source: Cancers (Basel). 2021 Sep 23;13(19):4749. doi: 10.3390/cancers13194749 (PMC8507538; doi:10.3390/cancers13194749)
Supplement: Supplementary file 1 [file cancers-13-04749-s001.zip › Supplementary table 1.pdf]

Table S1. Identification of cancer patients and comorbidities among the AP-HP warehouse

	ICD-10 Codes*	AMELI Codes**
Cancer		
Cancer	C00-C97;Z85;B21;D611;Y43;Z08;Z5101;Z511	All codes including ‘brachytherapy’, ‘irradiation’, ‘antitumoral drug‘, ‘malignant tumor surgery‘
Metastatic cancer	C77-C80	
Non-metastatic cancer	Cancer code without C77-C80	
Hematologic malignancies	C81-C96	
Digestive cancer	C15-C26	
Urologic cancer	C61-C68	
Gynecologic cancer	C51-58	
Pulmonary cancer	C34	
Breast cancer	C50	
Primary Central Nervous System cancer	C70-C72	
Head and neck cancer	C00-C14;C30-C32	
Thyroïd cancer	C73	
Melanoma	C43-C44	
Comorbidities		
Hypertension	I10-I13 ; I15	
Coronary heart disease or congestive heart failure	I11;I13;I20-I25;I50;Z95	
Cardiac arrythmia	I47-I49	
Diabetes mellitus	E10-E14	
Hyperlipidemia	E78	
Obesity	E66;T8550	
Chronic obstructive pulmonary disease	I27;J40-J47;J60-J70	
Smoker or history of smoking	F17;Z587;Z716;Z720	
Chronic kidney disease	I12-I13;N03;N05;N18-N19;N25;Z49;Z940;Z992	

*ICD-10 indicates International Classification of Diseases, 10th edition.
**CCAM, 63th edition
