# Supplementary material for: Clinical Characteristics, Care Trajectories and Mortality Rate of SARS-CoV-2 Infected Cancer Patients: A Multicenter Cohort Study
Source: Cancers (Basel). 2021 Sep 23;13(19):4749. doi: 10.3390/cancers13194749 (PMC8507538; doi:10.3390/cancers13194749)
Supplement: Supplementary file 1 [file cancers-13-04749-s001.zip › Supplementary table 2.pdf]

Table S2. COVID-19 treatments among patients with cancer and confirmed COVID 19.

**COVID-19 treatment (%)**	**All patients (N= 1148)**	**Patients who survived (N = 765)**	**Patients who died (N = 383)**	***P* value***
Admission to ICU	217 (18.9)	126 (16.5)	91 (23.8)	0.004
Mechanical ventilation	33 (2.9)	5 (1.1)	28 (4.1)	<0.0001
Corticosteroids (%)<br>*Missing data*	117 (10.2)<br>214 (18.6)	29 (6.2)<br>106 (22.7)	88 (12.9)<br>108 (15.8)	<0.0001
Immunotherapy<br>*Missing data*	42 (3.7)<br>214 (18.6)	11 (2.4)<br>107 (23)	31 (4.5)<br>107 (15.7)	0.002
Other**<br>*Missing data*	330 (28.7)<br>125 (18.7)	107 (23)<br>106 (22.7)	223 (32.7)<br>109 (16)	<0.0001

ICU: intensive care unit

* Groups were compared excluding missing data and using Pearson chi-square or Student T test as appropriate.

**: hydroxychloroquin, antivirals, macrolides
